# Modulating Effect of Ascorbic Acid on Transport-Induced Immunosuppression in Goats

**DOI:** 10.5402/2011/749753

**Published:** 2011-04-26

**Authors:** Ndazo Salka Minka, Joseph Olusegun Ayo

**Affiliations:** ^1^College of Agriculture and Animal Science, Division of Agricultural Colleges, P.M.B. 2134, Ahmadu Bello University, Mando, Kaduna, Nigeria; ^2^Department of Veterinary Physiology and Pharmacology, Faculty of Veterinary Medicine, Ahmadu Bello University, Zaria 810261, Nigeria

## Abstract

The effect of 12 h road transportation on some basic blood cells and the modulating role of ascorbic acid were investigated in 40 adult Red Sokoto goats during the hot dry season. The animals were divided into two groups, GI (experimental; *n* = 20) and GII (control; *n* = 20). Group 1 was administered with ascorbic acid (AA) *per os* at a dosage rate of 100 mg/kg body weight, while GII was given 10 mL of sterile water per goat. Forty minutes after the administration and loading, the goats were transported for 12 h. The result obtained in GII goats showed that loading, transportation, high ambient temperature (AT), and relative humidity (RH) encountered during transportation induced lymphopenia, neutrophilia, and eosinopenia, which can cause immunosuppression. In GI goats, the administration of AA prior to loading and transportation ameliorated the adverse effects of loading and transportation stress on neutrophil/lymphocyte ratio and eosinopenia of the goats.

## 1. Introduction

Road transportation of food animals is known to be stressful, resulting in increased morbidity and mortality. Transportation stress factors such as novelty of environment, deprivation of food and water, long journey duration, overcrowding, vehicle noise and vibration, and extreme environmental conditions are known to significantly weaken the animal's body resistance to diseases by depressing cellular and humoral immunity [1–3]. On a cellular level, it has been established that transportation stress induces leucocytosis, with associated lymphopenia, neutrophilia, and eosinopenia [3–6]. 

The effects of transport stress on the immune status of cattle, pigs, horses, and poultry are widely studied [1, 7], but in goats such studies are still limited in the available literature. Besides, little or no information is available on the effect of long-term journey duration on immune status of goats. Recently, transportation of goats has expanded worldwide due to the increased demand in goat meat and skin [3, 8, 9]. The few studies conducted on transportation conditions of goats are done in temperate regions of the world where livestock is predominantly raised intensively [3, 9, 10]. Factors affecting goats transported by road in tropical countries are high ambient temperature (AT), relative humidity (RH), and intense solar radiation occurring during the hot dry season of the year [3, 10]. In addition, the majorities of goats in tropical regions are reared under extensive management systems and are difficult to handle, and this may increase the level of stress encountered by goats during roundingup, handling, and loading.

The current management strategies towards alleviation of road transportation stress in animals included the use of analgesic, neuroleptics, electrolytes, or supplementing one or more dietary elements, before, during, or after transportation. Some of these agents are difficult to obtain and apply by the farmers; others are counterproductive, with great consequential effects on both animals and humans, while some have no effect and lack consistency and efficacy [7, 11–13]. Limited information is available on how to relieve road transportation stress in goats [3]. The identification of an additional agent that may be cheaper, nontoxic, with no withdrawal time, and easily administered would be of value in the field of animal transport and meat quality.

Neutrophil/lymphocyte (N/L) ratio has been used as reliable welfare indices in evaluating the immune status and adaptability of animals to various stress factors [4, 5].

There is a reasonable body of evidence supporting the use of ascorbic acid (AA) in reducing different kind of stresses in animals [2, 12, 14]. 

The present study investigated the effects of handling, loading, and 12 h of road transportation on some blood parameters in goats raised extensively during the hot dry season and suggested AA as an ameliorating agent against road transportation stress factors in goats.

## 2. Materials and Methods

### 2.1. Study Area and Meteorological Data

The experiment was performed at the Livestock Farm of the College of Agriculture and Animal Science, Ahmadu Bello University, Kaduna (11°10′N, 07°38′E), located in the Northern Guinea Savannah zone of Nigeria. 

The AT, RH, and sunshine duration were recorded at the experimental site at 07:00, 13:00, and 18:00  daily for seven consecutive days before and after transportation. Values of these parameters were also recorded at each of the twelve hours of the transportation period. The AT and RH were measured using a wet- and dry-bulb thermometer (Cocet, Shenzhen, Guangdong, China).

### 2.2. Animals and Management

Forty apparently healthy Red Sokoto goats, including males and nonpregnant females, two and a half to three years old and weighing between 23–25 kg served as subjects of the study. The goats were housed in a standard goat pen at a stocking rate of 1 m^2^/goat [9]. The goats were not restrained inside the pen. Each day, the goats were herded out and grazed on an improved natural pasture from 09:00 to 18:00,  and they were given access to drinking water *ad libitum*. 

### 2.3. Experimental Design

On transportation day, the goats were randomly divided into group 1 and II (GI and GII) comprising of 20 goats each. Blood samples were obtained from the goats during preloading, postloading, and posttransportation periods.

**Preloading Period**

Twenty minutes before loading and the administration of AA, ten goats from each group were colour marked and blood sampled at 07:00  to obtain preloading baseline values. Immediately after the first blood sampling, GI goats were administered orally with AA (Sigma Chemical, St. Louis, Mo, USA) at a dose of 100 mg/kg body weight [2, 3], dissolved in 10 mL of sterile water. At the same time, GII goats were administered orally with 10 mL of sterile water only.

**Postloading**

Thirty to 40 minutes after administration of AA and loading the goats before the start of the journey, another set of 10 goats from each group were blood sampled as above to evaluate stress response to handling and loading. This design was provided to eliminate the compounding effects of repeated handling and blood sampling on the goats [8]. The time lapse between pre- and postloading was allowed for the cortical level to peak up in circulation after imposing stresses, which usually takes 10–20 minutes [8].

**Posttransportation**

On completion of the journey, immediately after unloading, and subsequently 12 h, three and seven days after transportation period, all the goats were blood sampled.

### 2.4. Handling and Loading of Goats

The handling, loading, and transportation of the goats were carried out humanely in accordance with the guidelines governing animal transport welfare by road [15, 16]. The goats were stocked at a rate of 0.3 m^2^ per animal inside the vehicle [3, 8]. All the handling and loading were conducted between 06:00 to 07:00  (GMT + 1).

### 2.5. Vehicle Design and Journey Time

A standard Bedford, open pick-up van (made in England) was used for the journey. The floor of the van was provided with beddings (wood shavings) covered with rubber mats for secured footing. The journey commenced by 7:00  and was terminated at 19:00 . The vehicle travelled for 12 h with an average speed of 50 km/h on a single-lane road, from Kaduna to Makera (12°31′N, 06°11′E) town and from Makera back to Kaduna, covering a total distance of about 600 km. The vehicle was driven by two licensed drivers, and each drove the vehicle for six hours in a similar pattern. During the journey, the vehicle had four stopovers at veterinary and police control posts, and each stopover time was less than 5 minutes. In adherence to international transport welfare order, the goats were rested for an hour after 8 h transportation period. During the resting period, the vehicle was parked in a shade to avoid the direct effect of sunlight and rapid buildup of heat inside the vehicle. The goats were not fed or watered during the resting period [15]. 

### 2.6. Blood Analyses

About 5 mL of blood was collected from each goat at each period of blood sampling, into sterile test tubes with 0.14% anticoagulant (EDTA K3, Pty Ltd., Adelaide, SA, Australia). The collected blood was quickly kept in ice pack and taken to the laboratory where the blood samples were analysed for WBC and differential leucocyte counts [16]. Differential blood counts were performed through the preparation of thin monolayer blood smears placed on grease-free glass slides (Gold star micro slides, Chance Proper Ltd, UK). The smears were air dried and stained using the three-step stain for differentiation of morphological cell types (Bayer Corp., Diagnostic Division, Elkhart, IN, USA). Neutrophil : lymphocyte ratios were determined by using a 100/1.25 oil immersion objective, and 100 cells per slide were counted using the straight-edge method. The number of neutrophils was divided by the total number of lymphocytes to obtain the H/L ratio [16]. 

### 2.7. Statistical Analysis

Data were subjected to Student's *t*-test. ANOVA and Tukey's test were used to evaluate the effects of treatments (vitamin administration) and possible interaction between groups and period of sampling. The results are expressed as mean ± standard error of the mean (mean ± S.E.M.). Values of  *P* < .05  were considered significant.

## 3. Results

### 3.1. Meteorological Data during Transportation

The AT and RH recorded during transportation period, especially during the hot afternoon period, inside the vehicle rose with the duration of the journey from minimum values of 28.2°C and 65% for AT and RH at 07:00  to maximum values of 40.1°C and 80% for AT and RH at 14:00 , respectively. Thereafter, both the AT and RH decreased during the last hour (18:00 ) of the journey. The overall mean AT recorded during the journey was 37.2 ± 1.3°*C*, while that of the RH was 75.4 ± 1.8%.

### 3.2. Haematology

**Figure 1:**
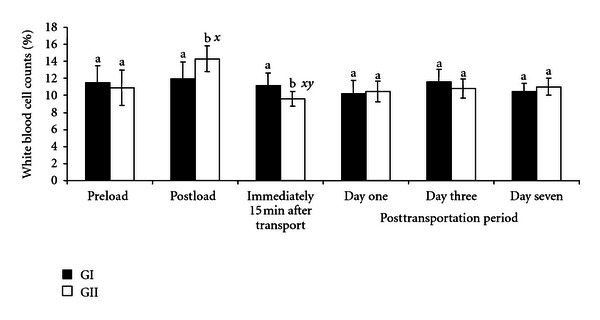
Changes in white blood cells counts (%) in GI and GII goats pre- and post-transportation. a, b = Bars with different alphabets are significantly (*P* < .05) different. *x* = *P* < .05 for pre-load; *y* = *P* < .05 for postload; *z* = *P* < .05  for immediately after transportation; *k* = *P* < .05 for one day after transportation. Pre- and postload, *n* = 10, posttransportation period,  *n* = 20.

The pre- and posttransportation WBC counts are shown in Figure 1. There was a significant (*P* < .05) increase in the values of WBC counts in GII goats from preloading value of 11.0 ± 2.1% to postloading value of 14.3 ± 1.5% and a decrease (*P* < .05) immediately after transportation to the value of 9.6 ± 0.9%. In the GI goats, the WBC counts were not affected by loading or transportation. 

**Figure 2:**
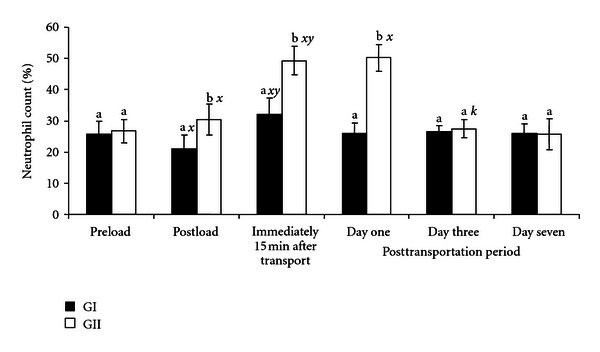
Changes in neutrophil counts (%) in GI and GII goats pre- and posttransportation. a, b = Bars with different alphabets are significantly (*P* < .05) different. *x* = *P* < .05  for preload; *y* = *P* < .05 for postload; *z* = *P* < .05 for immediately after transportation; *k* = *P* < .05 for one day after transportation. Pre- and postload, *n* = 10, posttransportation period, *n* = 20.

The neutrophil values recorded immediately after loading (30.3 ± 5.0%), transportation (49.3 ± 4.5%), and on day one after the journey (50.2 ± 4.2%) were significantly increased (*P* < .01) and were higher than the preloading value of 26.7 ± 3.7% recorded in GII goats. The neutrophil values obtained at the third and seventh day after transportation period did not differ from the preloading values. In GI goats administered with AA, the neutrophil count decreased significantly (*P* < .05) after loading but increased after the transportation. A day after transportation, the neutrophil value was restored to preloading value in GI goats (Figure 2).

**Figure 3:**
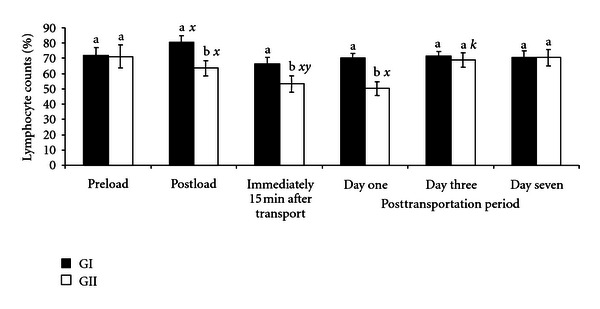
Changes in lymphocyte counts (%) in GI and GII goats pre- and post-transportation.. a, b = Bars with different alphabets are significantly (*P* < .05) different.  *x* = *P* < .05 for preload; *y* = *P* < .05 for postload;  *z* = *P* < .05 for immediately after transportation; *k* = *P* < .05 for one day after transportation. Pre- and postload, *n* = 10, posttransportation period, *n* = 20.

The changes observed in lymphocyte values are shown in Figure 3. In GII goats not administered with AA, there was a significant (*P* < .05) decrease in lymphocyte values from preloading value of 71.2 ± 7.5% to postloading and posttransportation values of 53.2 ± 5.3% and 50.2 ± 4.6%, respectively. In GI goats, the lymphocyte values increase after loading (80.5 ± 4.5%) over the preloading value of 71.8 ± 5.2%. In both GI and GII goats, the lymphocytes values were restored to the preloading count by the third day of posttransportation period.

**Figure 4:**
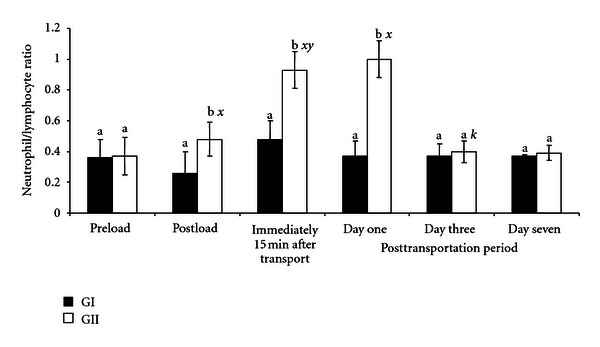
Changes in neutrophil/lymphocyte ratio in GI and GII goats pre- and posttransportation. a, b = Bars with different alphabets are significantly (*P* < .05) different. *x* = *P* < .05  for preload;  *y* = *P* < .05 for postload; *z* = *P* < .05 for immediately after transportation; *k* = *P* < .05 for one day after transportation. Pre- and postload, *n* = 10, posttransportation period, *n* = 20.

The pattern of changes in N/L ratio before and after transportation period is shown in Figure 4. In GII goats, the N/L ratio was significantly (*P* < .05) higher after loading and a day after transportation period (1.0 ± 0.12) compared to preloading value (0.37 ± 0.11), and the values were also higher (*P* > .05) than the corresponding N/L ratio recorded in GI goats. In GI goats, the increase in N/L ratio after loading and after transportation was not significantly higher (*P* > .05) than the preloading value (0.36 ± 0.12). Overall, the GII goats had higher (*P* < .05) N/L ratio after loading and transportation.

**Figure 5:**
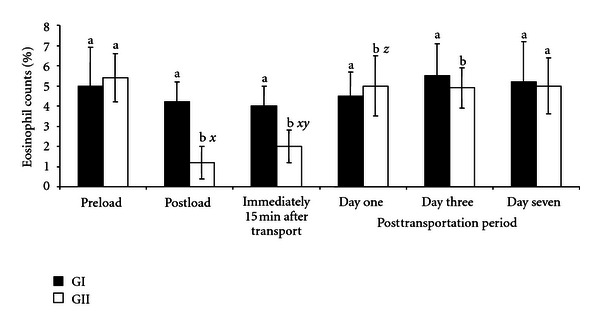
Changes in eosinophil counts in GI and GII goats pre- and posttransportation. a, b = Bars with different alphabets are significantly (*P* < .05) different. *x* = *P* < .05 for preload; *y* = *P* < .05 for postload; *z* = *P* < .05 for immediately after transportation; *k* = *P* < .05 for one day after transportation. Pre- and postload, *n* = 10, posttransportation period, *n* = 20.

The number of eosinophils in GII goats decreased (*P* < .05) rapidly after loading (1.2 ± 0.8%) and immediately after transportation (2.0 ± 0.8%) compared to the preloading value (5.4 ± 1.2%) and the values obtained in GI goats. The decrease in eosinophil value after loading and after transportation in GI goats administered with AA was not statistically different (*P* > .05) from the preloading value of 5.0 ± 1.9% (Figure 5). In both GII and GI goats, the eosinophil values were returned to baseline values a day after transportation. The monocyte and basophil counts were not affected by loading, transportation, and administration of AA in both GI and GII goats. 

## 4. Discussion

The AT and RH recorded in the present study were above the references range normal values of 20–30°C for AT and 60% for RH established for goats in the tropics [15]. The result suggested that the meteorological condition during the study period may not favour transportation of the goats. Such high AT and RH have been shown to induce heat stress which often results in the production of free radicals that causes oxidative damage of cells and tissues [17].

The initial increase in WBC counts after loading and their decrease after transportation in the GII goats, even though higher (*P* < .05) than the corresponding values recorded in GI goats, were still within the normal range values of 6–16% established for tropical goats [13, 18]. The change in WBC counts may be as a result of the presumed sudden release of glucocorticoids during handling and loading, which is responsible for the trafficking and release of WBC from the bone marrow [5, 19].

The result obtained in GII goats in the present study showed that handling, loading, and transportation significantly increased (*P* < .05) the value of neutrophil counts above preloading (25.8–26.7%) and the established baseline values of 27–46.8% for goats [18]. Similarly, the decreased lymphocyte counts were below baseline and the established values of 40–58.2% for goats [13, 18]. The initial rise in neutrophils and decrease in lymphocytes may be attributed to the effect of cortisol, released during the alarm stage of stress, which in this case was during handling and loading of the goats. This suggested that handling and loading induced both psychological and physiological stress to the goats and may constitute a serious welfare problem right from the onset of the journey. Catecholamines released during the alarm phase of stress have been reported to be the initial cause of neutrophilia and lymphocytosis [5]. Furthermore, the presumed corticosteroids released and the concomitant effects of high AT, RH, and other transportation stress factors encountered during the transportation period (resistance phase) contributed further to neutrophilia and lymphopenia. The results of the present study agree with similar findings in cattle [5], chickens [6], goats [8], and ostriches [20], who showed that transport-induced stress alters leucocytes profile. The fact that loading and transportation had no significant effect on the neutrophil and lymphocyte counts in GI goats demonstrated the ameliorating effect of AA against road transportation stress in goats.

The most common index of stress measurement from blood analysis is the N/L ratio [3, 4, 8], which has increased significantly (*P* < .05) above preloading baseline value of 0.4 and the recommended established values of 0.5–0.7 for goats in the tropics [13, 18]. The increase in N/L ratio in the present study was due to handling, loading, and transportation stress and is very predictive of disease. This is true especially for goats that are reared under the extensive management system and not accustomed to handling. The result indicated that the immune system of the goats was compromised as a result of handling, loading, and transportation, and the immune system remained suppressed up to the third day of posttransportation period. Leucocyte counts are important in evaluating prolonged effects of stress [8], and also the values reflect the effects of elevated corticosteroids in the circulation induced by stress [21]. 

The insignificant change in N/L ratio obtained in the GI goats administered with AA after loading showed, for the first time, that AA ameliorated the stresses induced by handling and loading on the lymphocytes and neutrophils of the goats. Handling and loading of animals, especially those that are reared under the extensive management system, are known to be the biggest stress factors and more stressful than the journey [22]. The results obtained during the posttransportation period showed that AA ameliorated the adverse effects of road transportation and posttransportation stress on N/L ratio, known to last for three to 7 days after the journey [4]. Apart from AA's inhibitory role on cortisol, AA has also been reported to be a chain-breaking antioxidant, involved in the prevention and restriction of free radical chain formation and propagation, consequently, protecting blood cells, including neutrophils and lymphocytes from oxidative damage [14, 19].

The number of eosinophil in GII goats decreased (*P* < .05) after loading and immediately after transportation far below the preloading value of 5% and the established baseline values of 4.7–5.5% for goats [18]. Three days after transportation, the preloading values were restored. This finding agrees with those of Nwe et al. [4] in transported native Japanese goats. However, Kannan et al. [9] failed to obtain any change in eosinophils of transported Spanish goats. Eosinopenia is known to be induced by physical and emotional stress, attributed to the elevated levels of plasma adrenaline and cortisol [4]. The result of the present study showed that the goats were subjected to prolonged physical and emotional stress during the 12 h journey, which was detrimental to their health. The mechanism of decrease in eosinophil numbers is uncertain, but it is believed that it is caused by intravascular lysis (steriod-induced apoptosis of eosinophils), decreased release from bone morrow, sequestration in organs such as the spleen and liver, and increased tissue migration [4]. It is worth noting that, in GI, goats such postloading and transportation decreases in eosinophil values were not statistically different (*P* < .05) and also from the preloading values. This may be due to the inhibitory role of AA on adrenaline and cortisol at the early stage of their release [6, 14]. 

There were no significant effects of transportation or administration of AA on monocyte and basophil counts. However, there was a slight but nonsignificant increase in monocytes of GI goats posttransportation period.

The overall result demonstrated that physical exertion and psychological insult often encountered during handling, loading, and transportation of goats, especially those reared under the extensive management system, were attenuated by the administration of AA 30–40 minutes before loading. Thus, the adverse effects of road transportation stress on blood immune cells were attenuated right from the onset of transportation by the administration of AA, a cheap, readily available, an easy to administered, and nontoxic vitamin.

## 5. Conclusions

In conclusion, the present finding demonstrated that handling, loading, and 12 h of road transportation were stressful to the goats and resulted in lymphopenia, neutrophilia, and eosinopenia, which are indicators of immunosuppression. The administration of AA prior to loading and transportation ameliorated the adverse effects of loading, transportation, and high AT and RH on the immune status of the goats and overcame the stress with less response.
